# Osteochondritis of the Distal Tibial Epiphysis

**DOI:** 10.1155/2012/629150

**Published:** 2012-11-06

**Authors:** Firass EL Hajj, Amer Sebaaly, Khalil Kharrat, Ismat Ghanem

**Affiliations:** Department of Orthopedic Surgery, Hotel Dieu de France Hospital, Alfred Naccache Street, Achrafieh, P.O. Box 166830, Beirut, Lebanon

## Abstract

Osteochondritis of the distal tibial epiphysis is a very rare entity. 9 cases have been described in 7 articles and 8 other cases have been mentioned in textbooks. This paper describes the 10th case of osteochondritis of the distal tibial epiphysis and summarizes the clinical and radiological presentations of the 9 other cases. The etiology of this entity is well debated in the literature. We believe that it results from a vascular abnormality in the distal tibial epiphysis associated with a mechanical stress (trauma, excessive overload, etc.). Since it is a self-limited disease, the prognosis is good and the younger the patient is the better the prognosis will be. In general, this entity responds well to conservative treatment.

## 1. Introduction

The first case of posttraumatic avascular necrosis of the distal tibial epiphysis was described by Siffert and Arkin in 1950 in an 11-year-old boy following a trimalleolar fracture [1]. This disease is very rare, and only nine cases have been reported so far. The purpose of this paper is to report the 10th known case of this disease, to summarize the other nine cases, to discuss its etiology, and to revise its clinical features, pathogenesis, and treatment.

## 2. Materials and Methods

### 2.1. Research Methodology

A systematic review of the literature has been made until the end of January 2012. We searched the following esearch engines: Pubmed, Cochrane Library and Science Direct, for “osteochondritis” OR “osteochondrosis” OR “avascular necrosis” AND “distal epiphysis” OR “tibia.” Finally, an internet-based general interest search engine, specifically Google (http://www.google.com/), was used to identify available sources that could potentially provide useful information, by using various combinations of the text words listed above. All of the articles found were included.

### 2.2. Case Presentation (See Figure 1)

**Figure 1:**
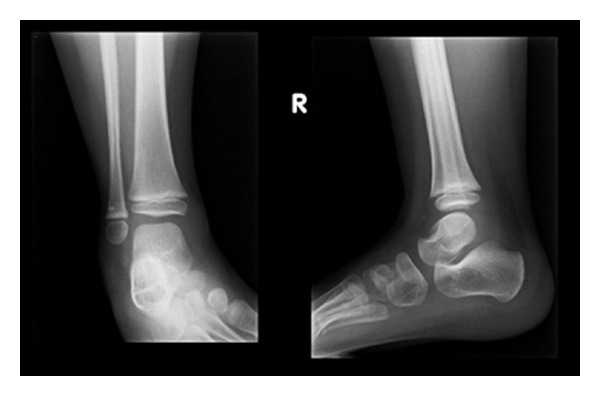
AP and lateral X-ray of the 4-year-old patient with normal findings.

A 6-year-old girl was referred to our department with a two-year history of swelling of the right ankle. In fact, two years ago the patient slipped while walking down the stairs. The X-rays of the right ankle were normal (Figure 1). One month later, she developed a swelling of the right ankle that was dealt with as being a post-traumatic swelling and treated with a cast immobilization for one month. The swelling did not resolve after cast removal but she was able to perform her daily activities normally without limping.

No X-rays were done during the two-year period until she was referred to our department.

On physical examination, ankle motion is preserved. There is a mild limb length discrepancy, the right limb being shorter than the left limb of less than 5 mm, but without limping. The circumference of the right ankle is increased in comparison with the left ankle. 

**Figure 2:**
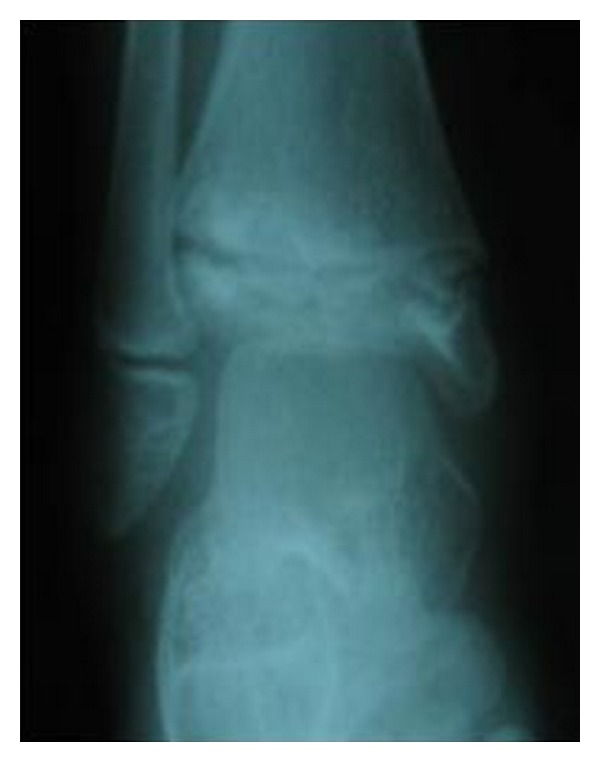
AP view of the ankle showing flattening and sclerotic appearance of distal tibial epiphysis. Irregular narrowing of the distal physis is seen with sclerosis rounding a lucency of the metaphyseal side of the growth plate. Note the normal appearance of the distal fibula growth plate.

**Figure 3:**
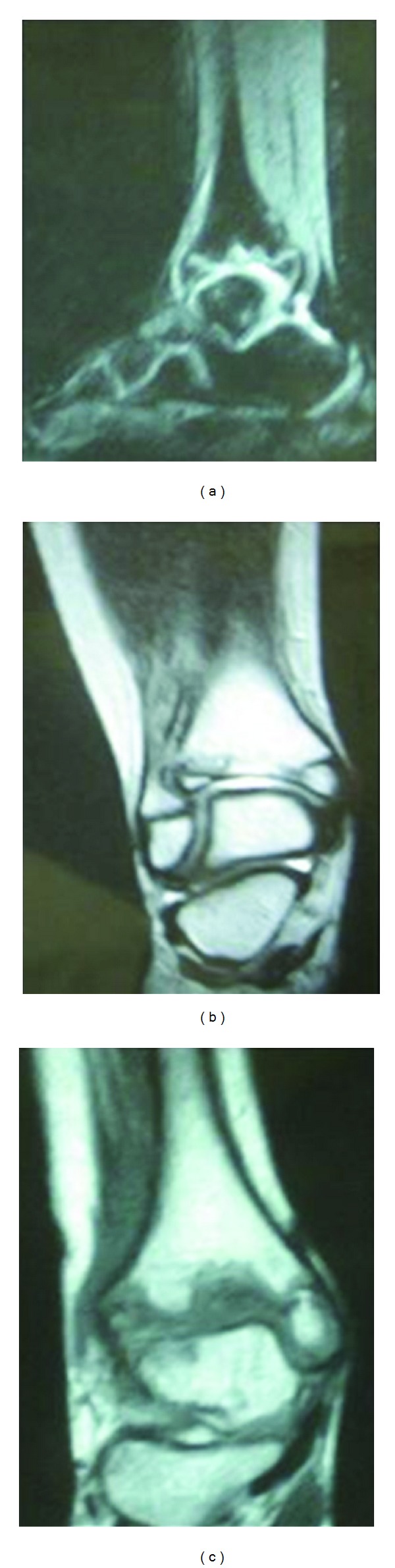
Sagittal T2W (a) and coronal T2W (b) and T1W (c) show total destruction of the epiphyseal centrum with erosion and bone marrow edema of the epiphysis with areas of fragmentation.

Laboratory work-up for infection and inflammation (white blood count (WBC), C-reactive protein (CRP), erythrocyte sedimentation rate (ESR), rheumatoid factor (RF), antinuclear antibodies (ANA), antidouble-stranded DNA, uric acid, T3, and thyroid stimulating hormone (TSH)) was normal and search for sickle cell was negative (Table 1). The X-rays were compatible with the diagnosis of distal tibial epiphysis avascular necrosis (Figure 2). They showed metaphyseal enlargement and irregularity of the distal tibial epiphysis. The MRI showed areas of fragmentation in the epiphysis (Figure 3). No treatment was given, and the child was subsequently lost to followup due to immigration.

## 3. Results

Seven articles (case reports) discussing nine cases of osteochondritis of the distal tibial epiphysis were found to which we add our case (Table 2). 

The ten cases studied consisted of 6 males and 4 females with a mean age of 7.6 years (range 3–13 years). 

In 2 cases, osteochondritis appeared after an ankle fracture [1, 6]. Four cases including ours had minor ankle trauma or practiced excessive sport activity; one of them had flexible pes planovalgus [2, 4, 5]. Three cases had neurological abnormalities and one case had congenital abnormality of the tibia without any documented traumatic event [3, 7].

All cases progressed spontaneously towards reconstruction of the distal tibial epiphysis with the exception of 3 cases. The first case [1] had an arthrodesis of the ankle 14 months after injury owing to persistent pain and nonunion of the fracture of the medial malleolus. The second [7] had congenital sensitive neuropathy and developed destruction of the ankle joint as in Charcot arthropathy; however, clinical and radiological signs found initially may have been early features of Charcot neurogenic joint. The third case [5] had minor clinical improvement without favorable radiological changes 22 months following injury. 

Eight cases were described in textbooks and were not published in scientific articles. Caffey in his textbook of pediatric radiology has listed 22 different areas in which osteochondritis has been described. He has seen only one case of osteochondritis of distal tibial epiphysis [8]. Weber et al. described a case but failed to demonstrate followup [9]. Rockwood et al. described a case with incomplete resolution at 9 months of followup [10]. Cummings reported a case of a patient who had significant joint stiffness and developed a valgus deformity secondary to collapse. After revascularization of the epiphysis, the ankle was realigned with a supramalleolar osteotomy, and 5 years later the patient had satisfactory function without pain [11]. Four other cases of osteochondritis of the distal tibial epiphysis have been reported after aggressive manipulation of clubfoot [11–13].

It is unclear from the ten cases in this study if young age at onset is associated with a better prognosis.

## 4. Discussion

Legg-Calve-Perthes disease of the proximal femoral epiphysis, Osgood-Schlatter disease of the tibial tubercle, and many other diseases have now come to be classified together as osteochondrosis because of the similarity of their clinical progression and radiologic presentation [3]. The epiphyses most commonly affected by osteochondrosis are those of the upper femur, the lunate bone, the navicular, and the head of the second metatarsal [4]. The distal tibial epiphysis is one of the most rarely affected epiphyses in the body.

Most of these diseases become apparent in the first two decades of life [5]. The clinical presentation of osteochondrosis often occurs after the bony nucleus appears in the epiphysis. For some years after this development, the epiphysis is mainly cartilaginous and, therefore, most susceptible to the osteochondrotic process. Once the bony epiphysis appears, it grows rapidly within the cartilage anlage. The cells of such a nucleus are thus very vulnerable and if there are supra-added mechanical pressures applied to this growing bone, the changes of osteochondrosis may occur. These changes are even more likely if there is a constitutional delayed appearance of ossification centers which is more frequent in males. This delay associated with the fact that boys are probably subjected to increased trauma and stress in early childhood may explain the higher prevalence of osteochondrosis among boys [14].

The etiology of osteochondritis of the distal tibial epiphysis is well debated in the literature and the exact mechanism of injury is still unknown. The two major factors that could be responsible for osteochondritis are the mechanical factor (trauma, overload, etc.) and the vascular factor (occlusion of vascular supply) or an association of both factors. The three cases of this study with neurological involvement may contribute to this mechanical factor and do not go against it. In the patient with spastic hemiplegia, abnormal load across the ankle joint is in concordance with the repetitive trauma history, common to all osteochondrosis. The case with sensory neuropathy and the one with myelomeningocele support the hypothesis that the lack of sensation may have contributed to the possible excessive load across the ankle joint as well.

The malleolar area is well supplied with blood by three arteries (posterior tibial artery, anterior tibial artery, and the peroneal artery). These vessels anastomose freely with one another and form arterial extraosseous networks below the corresponding malleoli [5]. In the case with congenital abnormalities of the tibia, both abnormal loading and possible congenital anomalous blood supply of the distal tibial epiphysis may be considered as possible causative factors.

In the 10 reported cases, 2 developed osteochondritis after ankle fractures, 4 had minor trauma, and 4 had congenital or neurological abnormalities without evidence of trauma. This can be explained by the fact that a developing epiphysis may be normal or it may have constitutional minor or major defects. Therefore, osteochondritis could be observed in a normal epiphysis subjected to extreme trauma, in a mildly dyschondrotic epiphysis subjected to more-than-usual stress and in a severely affected dyschondrotic epiphysis subjected to normal stress [14].

We believe that the association of vascular, traumatic, and constitutional features described above predisposes the distal tibial epiphysis to injury and subsequent osteonecrosis and can explain the rarity of the osteochondritis of the distal tibial epiphysis. 

Symptoms and signs observed in osteochondritis of the distal tibial epiphysis are similar to those observed in osteochondritis in other sites. The patient complains of pain in the affected site. Physical examination shows localized tenderness, limited ankle movement, swelling, and reactive effusion in the adjacent joint.

Few months after the aggression, the radiologic abnormalities become evident. Three phases are observed: the densification phase, the fragmentation phase, and finally the reconstruction phase. The total duration of these phases is generally 3 to 5 years.

In general, osteochondritis of the distal tibial epiphysis is managed with rest to avoid loading while maintaining a good range of movement of the ankle joint. Drilling the area to stimulate revascularization is used by some authors without any scientific evidence of efficacy [4]. Because osteochondritis is a self-limited disease it is considered a benign condition which responds well to conservative treatment. In the case presented by Robertson et al., it is of interest that regeneration took place in spite of the fact that the child continued to bear weight and that the joint was immobilized for only 2 months beginning four months after the original injury [2].

In some cases, there may be an alteration in the shape of the affected region after reossification has occurred, so a displacement osteotomy may improve the containment of the affected joint [4].

The prognosis is determined by the mechanism of injury, the severity of involvement and, in theory, the growth potential of the involved area and the young age of the patient at onset. However, we were unable to determine in this study any influence of young age at onset over the final outcome.

## 5. Conclusion

Osteochondritis of the distal tibial epiphysis is a very rare entity. In this paper, we have reviewed the only 10 known cases. The mechanism of this disease could be the association of a congenital anomalous blood supply of the distal tibial epiphysis and a severe or repetitive trauma if the epiphysis is constitutionally normal or a minor trauma if the epiphysis has minor or major constitutional defects. It is a self-limited disease and the majority of the cases had a good evolution with conservative treatment.

## Figures and Tables

**Table 1:** Laboratory findings.

Test ordered	Finding	Normal range
WBC	6500	5.500–15.500
ESR	3 mm/hour	0–20 mm/hour
CRP	<5	<5
RF	<20 UI/mL	<20 UI/mL
ANA	Negative (ELISA)	
Anti ds-DNA	<40	<40
Uric acid	3.9 mg/dL	2.0–6.0 mg/dL
T3	219 ng/dL	100–260 ng/dL
TSH	3.72 *μ*UI/mL	0–10 *μ*UI/mL
Sickle cells	Negative	

**Table 2:** Comparison in clinic, diagnosis, and management of different cases in the literature.

Author	Publication year	Number of cases	Age (years)/sex	Etiology	Treatment of etiology	Clinical presentation of AVN	Radiology	Treatment and evolution of AVN
Siffert and Arkin [1]	1950	1	11/M	Comminuted trimalleolar ankle fracture (Salter Harris IV)	(i) Reduction under general anesthesia (ii) Cast stabilization for 2 months (iii) Gradual weight bearing after cast removal	(i) Symptoms started shortly after cast removal (ii) *13 months* after injury: pain, swelling, restriction of motion	(i) X-ray (*13 months*): nonunion of the medial malleolus and epiphysis irregular, compressed, and fragmented	(i) Ankle arthrodesis *14 months* after injury owing to persistent pain and nonunion

Robertson [2]	1964	1	3/M	Left ankle trauma without radiological abnormality		(i) *4 months* after trauma: pain, medial ankle swelling	(i) X-ray (*4 months*): flattening and sclerosis of the distal tibial epiphysis (ii) X-ray (*10 months*): new bone formation and early regeneration	(i) Immobilization with a below knee cast for 2 months (ii) *5.5 years* after injury: no clinical abnormality

Hassler et al. [3]	1960	2	Case *1*: 7,5/F	Known to have: (i) Congenital anterior bowing of the tibia (ii) Absence of proximal one-third of the fibula		(i) Started walking at *3.5 years * (ii) At *7.5 years*: clinical widening of ankle was noted	(i) X-ray (*age 7.5 years*): fragmentation of distal tibial epiphysis (ii) X-ray (*2 years* after diagnosis): reformation of bone trabeculae in the epiphysis	(i) Walks with a knee-to-ankle brace
			Case *2*: 3/M	Known to have: (i) Mild spastic right hemiplegia (ii) Valgus deformity of right foot (iii) Mild recurrence of left metatarsus adductus deformity	Brace for valgus deformity	(i) Prominence of left medial malleolus (ii) Tendency of the left foot to go into varus	(i) X-ray (*age 3 years*): No ossification of distal tibial epiphysis (ii) X-ray (*age 5 years*): Epiphysis flattened and fragmented (iii) X-ray (*age 8 years*): Normal	(i) Brace for 1 year to control the varus deformity (ii) The patient walks in a regular shoe at *8 years* of age

Klein et al. [4]	2008	1	12/M	(i) Known to have: flexible pes planovalgus (ii) Played football and kicked with his right foot		(i) Tenderness and swelling on the medial malleolus *6 weeks* after playing football	(i) X-ray (*6 weeks* after sport): fragmentation of the right medial malleolus (ii) X-ray (*10 weeks* after sport): signs of repair of the epiphysis (iii) MRI (*same time*): soft tissue and bone-marrow oedema at the medial malleolus	(i) Conservative treatment (ii) *10 weeks* (after sport): no symptoms

Holland et al. [5]	1993	1 (bilateral)	13/F	School sport tournament *10 months* before presentation		(i) Bilateral ankle pain (ii) Unable to practice sports (iii) Mild swelling and a decreased range of motion of both ankles, especially of the left one	(i) X-ray: bilateral sclerosis, fragmentation, and collapse of the lateral part of the distal tibial epiphyses and the adjacent metaphyses	(i) Restriction of activity with arch supports (ii) *1 year * *later* her complaints were slightly diminished. (iii) The radiographs (*1 year*) showed no significant changes

Kennedy and Weiner [6]	1991	1	12/M	(i) Salter IV fracture of the right medial malleolus (ii) Salter II fracture of the distal fibula	(i) Closed reduction in the ER (ii) ORIF with 3 pins (iii) Pins removed at *6 weeks *		(i) X-ray (*6 weeks*): some persistence of the malleolar fracture and the tibial epiphysis unusually sclerotic (ii) X-ray (*20 weeks*): increased epiphyseal density (iii) X-ray (*49 weeks*): reossification of the tibial epiphysis. (iv) Bone scan (*18 months*): showed revascularization of the epiphysis	(i) Short leg cast for *8 weeks* (ii) Touch-down weight bearing *8 weeks* after cast removal (iii) Clinical improvement (iv) Nonprotected walking at *20 weeks* *18 months* after injury: clinically asymptomatic

Gascó et al. [7]	2010	2	Case *1*: 4/F	(i) Known to have congenital sensitive neuropathy (ii) Swelling of right ankle started *2 years ago *	(i) Below knee cast for *1 month* (traumatic suspicion) but persistence of swelling after its removal	(i) At presentation: ankle swelling and reduced ROM with subtalar stiffness (ii) Gait normal with no limb length discrepancy	(i) X-ray (*at presentation*): increase in density and sclerosis with height reduction of the distal tibial epiphysis (ii) MRI: hypointense band located in the ossification nucleus of the distal epiphysis (iii) X-ray *after 1 year*: signs of reossification with widening of the metaphysis and epiphysis (iv) X-ray (*5 years*): destruction of the ankle joint as Charcot arthropathy	(i) *At presentation*: ankle-foot orthosis to prevent postural bad habit for a period of 3 months (ii) At *2 years*: mild ankle swelling with normal ROM (iii) At *5 years*: reduced ROM. There is also a varus deformity of the hind foot and subtalar joint stiffness and a 1.5-cm limb length discrepancy The patient still use an orthosis for ankle protection
			Case *2*: 5/M	Known to have myelomeningocele and developmental dysplasia of the right hip operated at 3 years of age: Dega acetabuloplasty and varus derotation osteotomy of the femur		Right ankle swelling for 2 weeks	(i) X-ray at presentation: reduction in the tibial epiphyseal height and an increase in bone density (ii) X-ray (*after * *5 * *years* of follow-up): recovery of the height of the epiphysis	(i) Rest for 6 weeks After *5 years* of followup: ROM similar to other side. Leg length discrepancy was less than 1.5 cm caused by the pathology of the ankle and that of the hip

Our case	2011	1	6/F	Right ankle trauma without radiological abnormality		Right ankle swelling 1 month after trauma Right limb shorter than the left limb of less than 5 mm	(i) X-rays (during *the first 2 years* of evolution): compatible with the diagnosis of AVN (ii) X-ray (after *2 years* of evolution): metaphyseal enlargement and irregularity of the epiphysis (iii) MRI: areas of fragmentation in the epiphysis	(i) No treatment was given. (ii) Clinical and radiological followup at regular intervals
